# Supplementary figures and images for: GSEA–SDBE: A gene selection method for breast cancer classification based on GSEA and analyzing differences in performance metrics
Source: PLoS One. 2022 Apr 26;17(4):e0263171. doi: 10.1371/journal.pone.0263171 (PMC9041804; doi:10.1371/journal.pone.0263171)

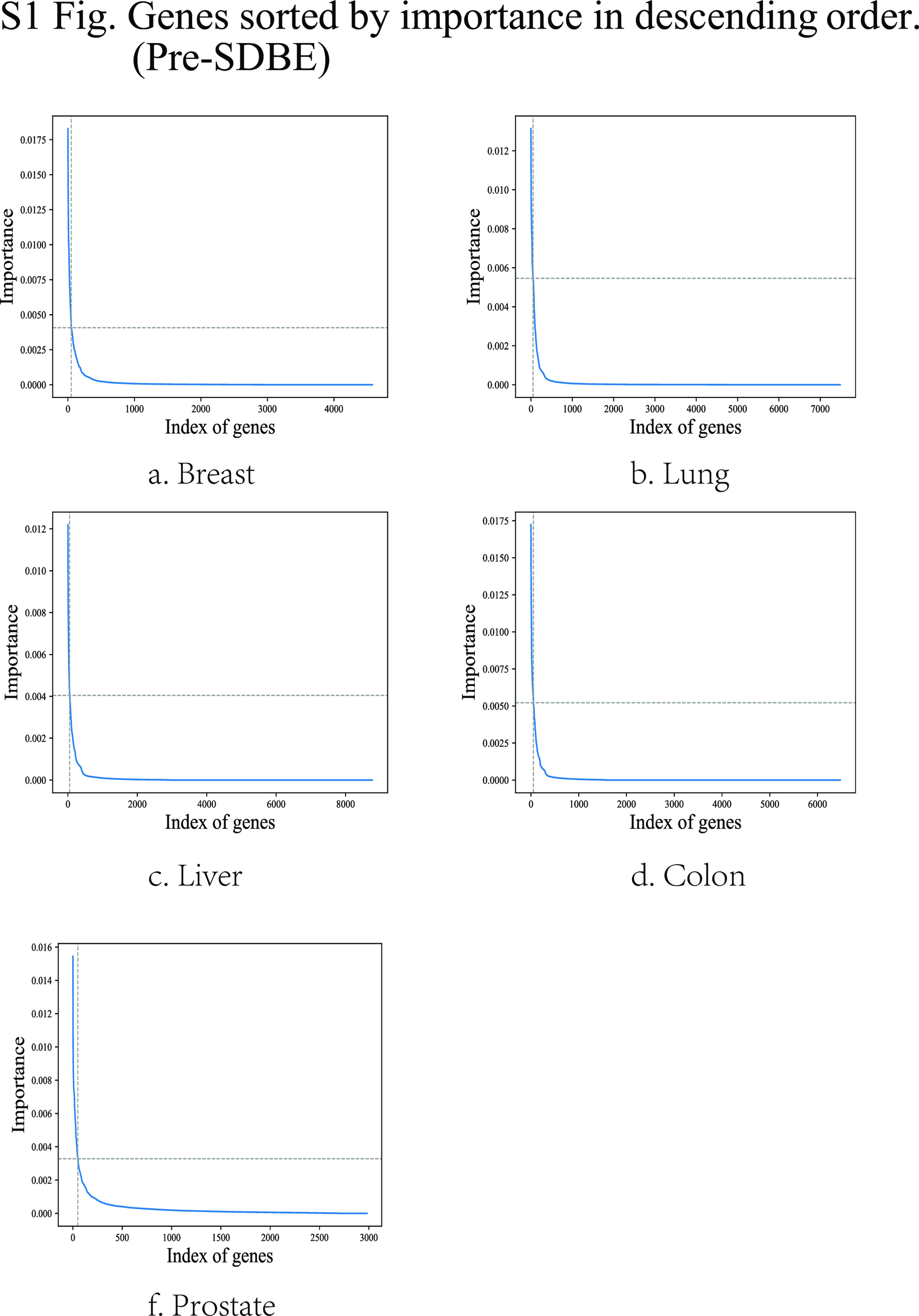

Supplement: S1 Fig — (TIF) [file pone.0263171.s001.tif]

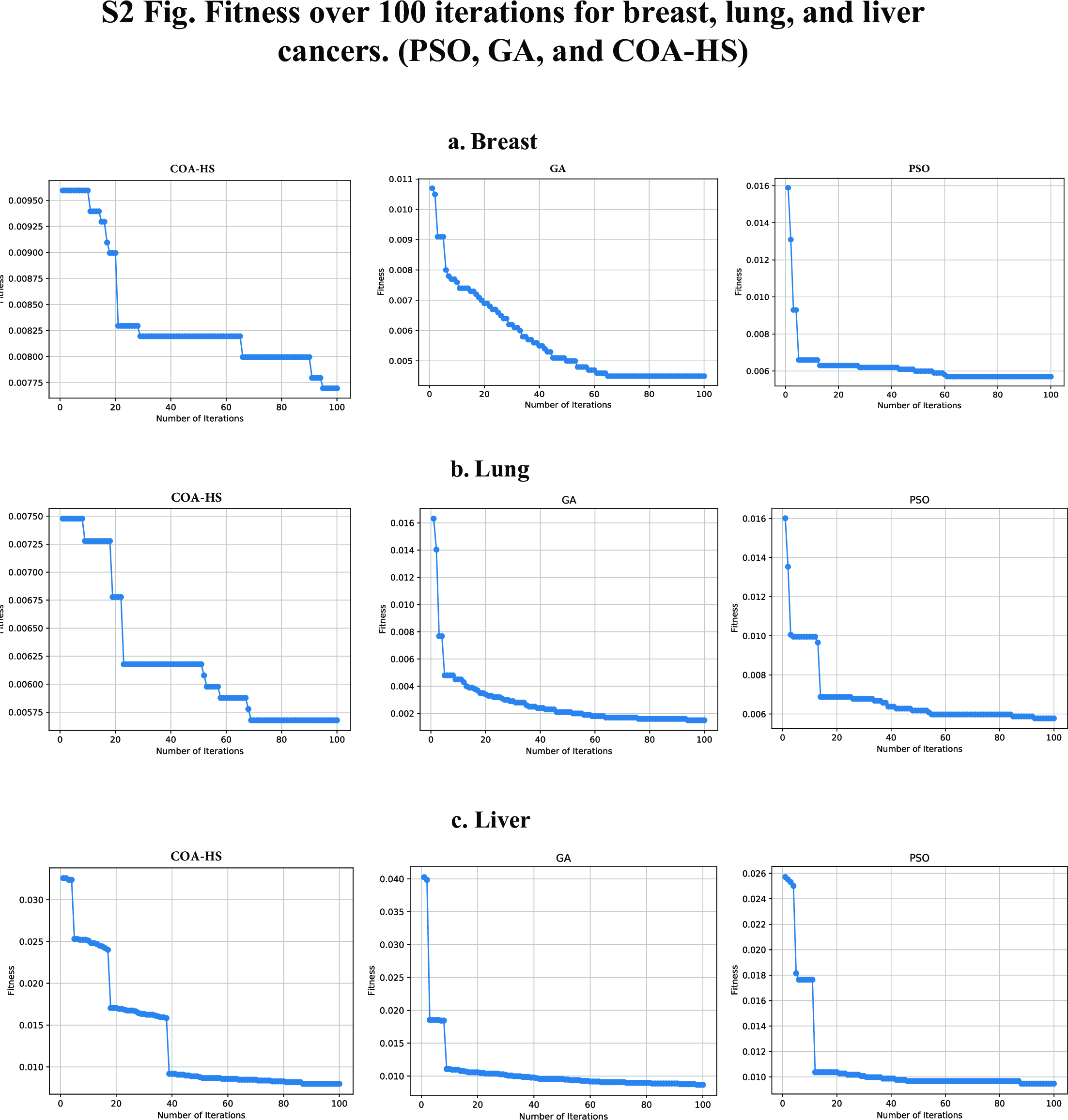

Supplement: S2 Fig — (TIF) [file pone.0263171.s002.tif]

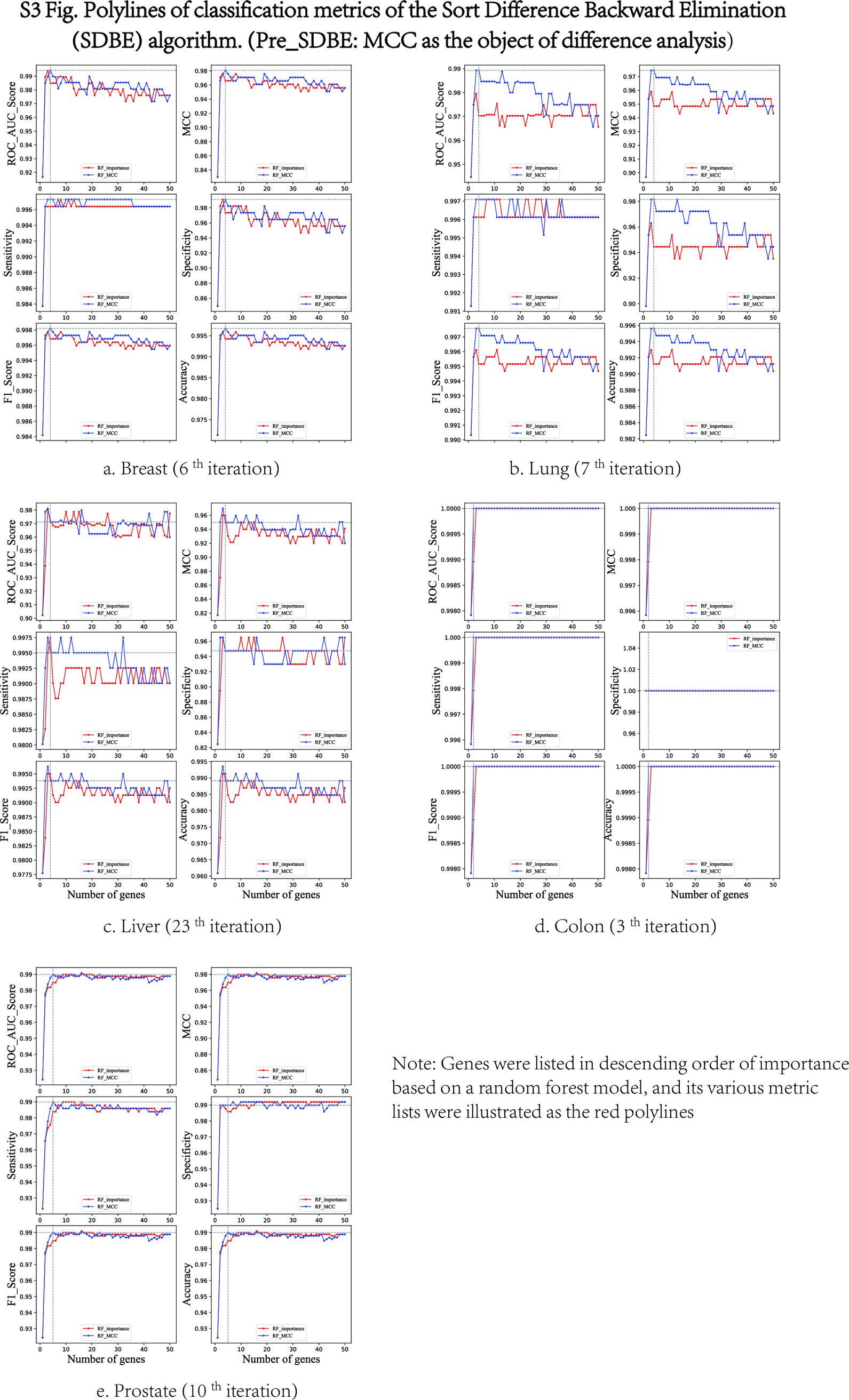

Supplement: S3 Fig — (TIF) [file pone.0263171.s003.tif]

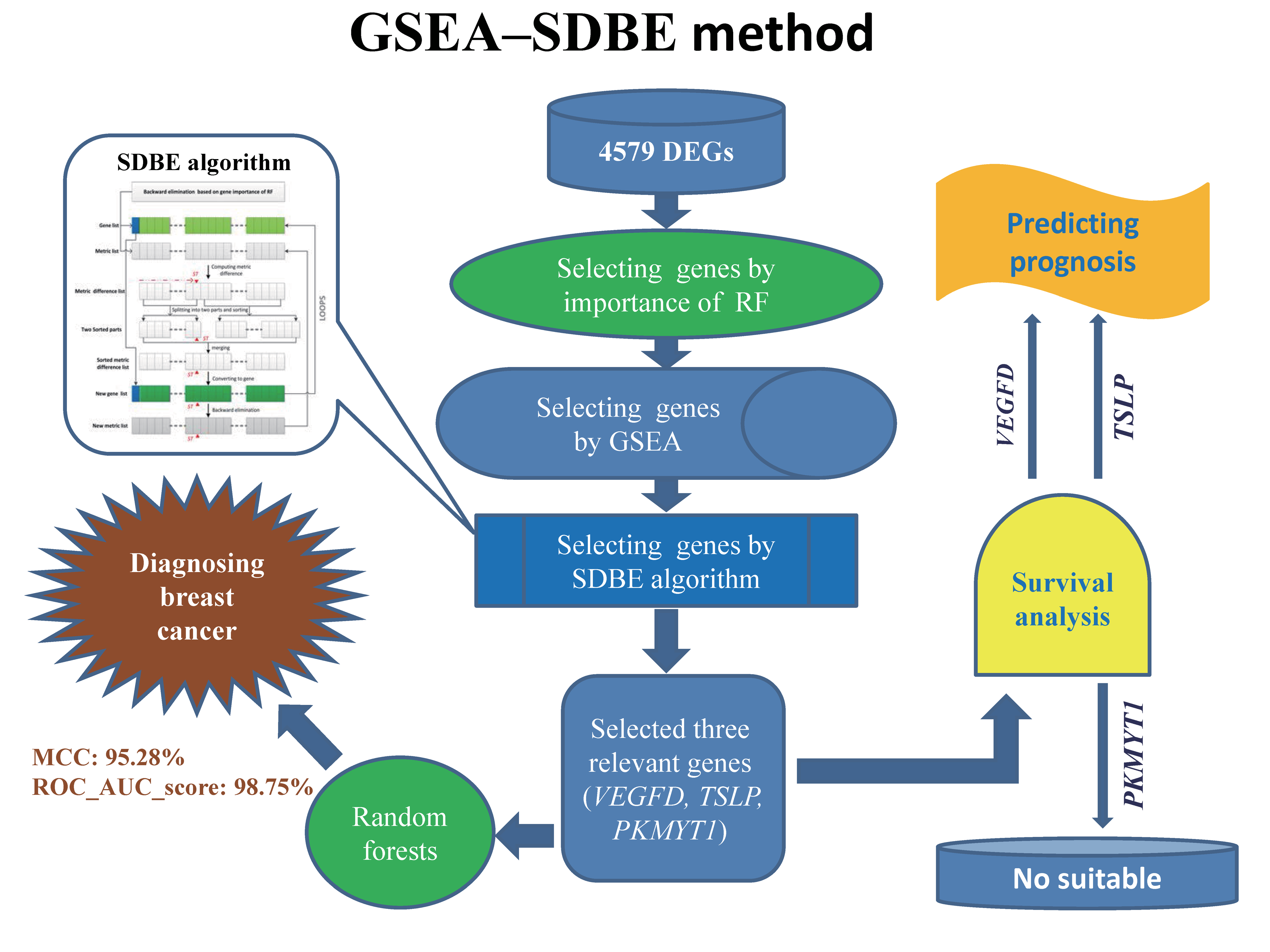

Supplement: S1 Graphical abstract — (TIF) [file pone.0263171.s010.tif]
