# Supplementary material for: GSEA–SDBE: A gene selection method for breast cancer classification based on GSEA and analyzing differences in performance metrics
Source: PLoS One. 2022 Apr 26;17(4):e0263171. doi: 10.1371/journal.pone.0263171 (PMC9041804; doi:10.1371/journal.pone.0263171)
Supplement: S6 Table — (DOCX) [file pone.0263171.s009.docx]

| **Algorithm** | **#Genes** | Selected genes | **classification metrics** | Program time |
| --- | --- | --- | --- | --- |
| MRMR**+PSO ^a^** | 30 | *ANAPC11, METTL26, C1orf35, AC010336.2, AC138696.2, AZU1, AL390719.2, SCX, LIPE-AS1, SPC24, RECQL4, IER5L, FAM110A, MRPL24, PAK3, CLDN7, SRMS, IGSF9, AC010761.6, HOXC10, HDAC11, SIPA1L3, JARID2-AS1, IGFLR1, PMAIP1, C5orf38, CERS1, PDGFD, THSD1, COL24A1* | accuracy_score: [0.9673024523160763], precision_score: [0.9938461538461538],  recall_score: [0.96996996996997],  f1_score: [0.9817629179331306],  roc_auc_score: [0.9555732202791026],  FPR: [0.058823529411764705],  TNR: [0.9411764705882353],  MCC: [0.8298191109054869] | MRMR: 46.2 h;  PSO: 27.6200 s |
| MRMR**+GA ^a^** | 18 | *AL353708.3, ZC3H3, MXD3, TRPM2-AS, MRPL55, LIPE-AS1, AC137630.3, MRPL14, MRPL24, PAK3, IGSF9, GRIK1-AS1, GPRASP1, HDAC11, IGFLR1, AC015908.2, LRRC2, THSD1* | accuracy_score: [0.9782016348773842], precision_score: [1.0],  recall_score: [0.975975975975976], f1_score: [0.9878419452887537], roc_auc_score: [0.987987987987988], FPR: [0.0],  TNR: [1.0],  MCC: [0.8888620759505264] | MRMR:46.2 h; GA: 39.8088 s |
| MRMR**+COA-HS ^a^** | 11 | *METTL26, C1orf35, AC006077.2, C20orf204, PFDN6, OXLD1, MMP17, LIPE-AS1, PYCR3, EXOSC4, MRPL14* | accuracy_score: [0.9836512261580381], precision_score: [0.9969604863221885], recall_score: [0.984984984984985], f1_score: [0.9909365558912387], roc_auc_score: [0.9777866101395513], FPR: [0.029411764705882353],  TNR: [0.9705882352941176],  MCC: [0.9093598979140415] | MRMR:46.2 h ; COA-HS:83.5140 s |

**S6 Table. Classification performance information of three advanced algorithms (PSO, GA, and COA-HS) for three cancer datasets.**

**a. Breast**

**b. Lung**

| **Algorithm** | **#Genes** | Selected genes | **classification metrics** | Program time |
| --- | --- | --- | --- | --- |
| MRMR**+PSO ^a^** | 29 | *RXFP1, AC245517.5, AC245060.5, HOXC12, TMEM63C, FAM25A, AC125611.2, U62317.1, SLA, TNKS2-AS1, NCF1B, HMGCLL1, FMO2, NPR1, CHRM1, ESCO2, SNORD51, YEATS2, SLC12A8, RASSF10-DT, AC010976.2, MDFI, AL355472.3, RPS27P16, CPA4, CACNA2D2, IGHA2, DLL4, C12orf66],* | accuracy_score: 0.9767441860465116, precision_score: 1.0,  recall_score: 0.9743589743589743, f1_score: 0.9870129870129869, roc_auc_score: 0.9871794871794872, FPR: 0.0,  TNR: 1.0,  MCC: 0.882885711444596 | MRMR:49.5 h ;  PSO: 25.46 s |
| MRMR**+GA ^a^** | 15 | *POSTN, IGKV3D-11, TMEM63C, AC245060.2, SLA, AGTR2, RNU1-36P, NCF1B, EME2, AC013410.2, MT1M, YWHAZP6, FMO2, C2CD4D, AC112206.4],* | accuracy_score: 0.9825581395348837, precision_score: 1.0,  recall_score: 0.9807692307692307, f1_score: 0.9902912621359222, roc_auc_score: 0.9903846153846154, FPR: 0.0, TNR: 1.0,  MCC: 0.9087964189843392 | MRMR: 49.5h;  GA: 31.97 s |
| MRMR**+COA-HS ^a^** | 8 | *P3H3, RXFP1, TMEM213, HOXC12, TMEM63C, PAICS, H4C11, IGKV6-21* | accuracy_score:0.9796511627906976, precision_score: 1.0,  recall_score: 0.9775641025641025, f1_score: 0.9886547811993517, roc_auc_score: 0.9887820512820513, FPR: 0.0,  TNR: 1.0,  MCC: 0.8956025228929602 | MRMR:49.5 h ; COA-HS:86.87 s |

**c. Liver**

| **Algorithm** | **#Genes** | Selected genes | **classification metrics** | Program time |
| --- | --- | --- | --- | --- |
| MRMR**+PSO ^a^** | 24 | *ZNF222, ZFAS1, NAE1, PIGT, SAE1, AC006213.3, MRPL13, EIF3G, BRF2, DHX37, ZNF829, AC002398.1, DAPK2, MED27, STMN3, NOP2, RPS12, PDCD2L, ZNF529-AS1, DYRK1B, TUBB2B, RPL21P134, FBXW9, DDX10],* | accuracy_score: 0.8571428571428571, precision_score: 1.0,  recall_score: 0.8373983739837398, f1_score: 0.911504424778761, roc_auc_score: 0.9186991869918699, FPR: 0.0,  TNR: 1.0,  MCC: 0.6202826809089065 | MRMR:27.5 h ; PSO:8.15 s |
| MRMR**+GA ^a^** | 16 | *FJX1, NAE1, PIGT, SRM, MRPL13, RPL18AP3, VASP, AC127070.1, IRGQ, AC095057.3, STMN3, EIF2S2, KMT5C, DOC2B, NUMBL, TSEN2],* | accuracy_score: [0.8928571428571429], precision_score: 1.0,  recall_score: 0.8780487804878049, f1_score: 0.9350649350649352, roc_auc_score: 0.9390243902439024, FPR: 0.0,  TNR: 1.0,  MCC: 0.6829812695817082 | MRMR:27.5 h GA:11.60 s |
| MRMR**+COA-HS ^a^** | 9 | *PIGT, HIKESHI, RBM28, NOMO1, MEGF8, TUBB2B, RPL21P134, NUMBL, TSEN2],* | accuracy_score:0.9142857142857143, precision_score: 1.0,  recall_score: 0.9024390243902439, f1_score: 0.9487179487179488, roc_auc_score: 0.9512195121951219, FPR: 0.0,  TNR: 1.0,  MCC: 0.7273348470781456 | MRMR:27.5 h ; COA-HS:18.05 s |

**Note:** FPR: false positive rate; TPR: sensivity; TNR: specificity; ROC_AUC_score: computed area under the receiver operating characteristic curve from prediction scores; MCC: matthews correlation coefficient; h: hour; s: second.
