# Supplementary material for: GSEA–SDBE: A gene selection method for breast cancer classification based on GSEA and analyzing differences in performance metrics
Source: PLoS One. 2022 Apr 26;17(4):e0263171. doi: 10.1371/journal.pone.0263171 (PMC9041804; doi:10.1371/journal.pone.0263171)
Supplement: Attachment — Submitted filename: Response to Reviewers.docx [file pone.0263171.s011.docx]

**List of Responses**

**Dear Editors and Reviewers:**

Thank you for your letter and for the reviewers’ comment concerning my manuscript entitled “GSEA–SDBE: A gene selection method for breast cancer classification based on GSEA and analyzing differences in performance metrics” (ID: PONE-D-21-09636). Those comments are all valuable and very helpful for revising and improving my paper, as well as the important guiding significance to my researches. I have studied comments carefully and have made correction which I hope meet with approval. Revised portion are marked in red in the paper. The main concerns in the paper and the responds to the reviewer’s comments are as flowing:

**Responds to the reviewer’s commants:**

**Reviewer #1:**

1. **Response to comment:** ( I think if the author provides a practical flowchart for readers it would be more applicable.)

**Response:** I am very sorry for my negligence. The revised practical flowcharts are as follows.

**Modified to**

**Fig. 1. Procedure of the Sort Difference Backward Elimination (SDBE) algorithm.**

**Modified to**

**Fig 2. Gene selection procedure in the GSEA–SDBE method.**

2. **Response to comment :** (Also please provide some information about survival of the patients in the results section.)

**Response:** I am very sorry for my negligence of providing some information about survival of the patients. So I added the S4 table, which provided this information.

**Reviewer #2:**

1. **Response to comment** :( The paper is missing a comparison with other state-of-the-art methods for gene selection.)

**Response:** I am very sorry for my negligence. The performance comparison with other models as follows.

Performance comparison with other models

The Pre-SDBE is a simplified version of the GSEA-SDBE method, which does not contain the use of GSEA to filter out genes weakly associated with or unrelated to cancer. The other three state-of-the-art-methods [7], the genetic algorithm (GA), the particle swarm optimization (PSO) algorithm, and the cuckoo optimization algorithm and harmony search (COA-HS), also does not filter out DEGs that have no biological significance for cancer. Therefore, the performance of the Pre-SDBE can be compared with these three algorithms which use relevant genes selected by the minimum redundancy and maximum relevance (MRMR) as input data.

In the Pre-SDBE algorithm, the top 50 relevant genes from DEGs were selected in the step that selected genes by their importance based on a random forest model (S1 Fig). Next, these selected genes were fed into SDBE algorithm to pick the best genes while maintaining the highest accuracy. The number of iterations in SDBE algorithm was respectively set at 3 and 10 for the colon and prostate cancer dataset (S5 Table).

**Table 5. Information on the microarray datasets used in this comparison**

| **Microarray dataset** | **Number of genes** | **Number of samples** | **Normal** | **Tumor** |
| --- | --- | --- | --- | --- |
| Prostate [32] | 12,600 | 102 | 50 | 52 |
| Colon [33] | 7,457 | 62 | 22 | 40 |

In the case of the colon cancer dataset, the Pre-SDBE method outperformed other algorithms, reaching 100% accuracy with the fewest number of genes (2 genes) compared to all other algorithms (S2 Fig,). For the prostate cancer dataset, the Pre-SDBE method could also achieve accuracy (98.99%) with the fewest genes (5 genes) (Table 6 and S3 Fig).

**Table 6. Performance comparison of Pre-SDBE with three models for two microarray datasets**

| **Algorithm** | **Colon** | | | | **Prostate** | | | |
| --- | --- | --- | --- | --- | --- | --- | --- | --- |
|  | **#Genes** | **AC** | **SE** | **SP** | **#Genes** | **AC** | **SE** | **SP** |
| Pre-SDBE | 2 | 1.0000 | 1.0000 | 1.0000 | 5 | 0.9899 | 0.9899 | 0.9899 |
| PSO [7] | 11 | 0.9642 | 0.8580 | 1.0000 | 19 | 0.9804 | 0.9180 | 1.0000 |
| GA1 [7] | 14 | 0.9516 | 0.8460 | 1.0000 | 28 | 0.9804 | 0.9180 | 1.0000 |
| COA-HS [7] | 5 | 1.0000 | 1.0000 | 1.0000 | 5 | 1.0000 | 1.0000 | 1.0000 |

AC:Accuracy; SE:Sensitivity; SP:Specificity; Number of selected genes: #Genes.

2. **Response to comment :** (A discussion on the computational complexity of the method after comparison with the other methods is missing.)

**Response:** I am very sorry for my negligence. The discussion about computational complexity as follows.

In the Pre-SDBE method, the top 50 relevant genes were first selected by their importance based on a random forest model, and its number was only half of the number of genes selected using MRMR [7]. Perhaps fewer genes could be selected as the input to the SDBE algorithm to reduce time-consuming. However, whether it is GSEA-SDBE method or its simplified version (Pre-SDBE), 10-fold cross-validation is the main factor leading to time-consuming.

**Some minor comments**

1. **The paper needs language corrections.**

**Response:** I am very sorry for my incorrect writing. So, I have seriously corrected the language. Please check the revised paper.

2. **References are not coherent. Some references are missing authors' names (et al. should not be in the bibliography)**

**Response:** I am very sorry for my incorrect writing and revised the references as follows.

**References**

**1.** Hartmaier R, Albacker LA, Chmielecki J, Bailey M, He J, Goldberg ME, et al.. High-throughput genomic profiling of adult solid tumors reveals novel insights into cancer pathogenesis. Cancer Research. 2017;77:2464–2475. doi: 10.1158/0008-5472.CAN-16-2479.

**2.** Giovannantonio MD, Harris BH, Zhang P, Kitchen­Smith I, Xiong L, Sahgal N, et al. Heritable genetic variants in key cancer genes link cancer risk with anthropometric traits. Journal of Medical Genetics. 2020;0:1–8. doi: 10.1136/jmedgenet-2019-106799.

**3.** Dı´az-Uriarte R, Andre´s SAd. Gene selection and classification of microarray data using random forest. BMC Bioinformatics. 2006;7(3):1–13. doi: 10.1186/1471-2105-7-3.

**4.** Pok G, Liu J-CS, Ryu KH. Effective feature selection framework for cluster analysis of microarray data. Bioinformation. 2010; 4(8):385–389. doi: 10.6026/97320630004385.

**5.** Xie J, Wang C. Using support vector machines with a novel hybrid feature selection method for diagnosis of erythemato-squamous diseases. Expert Syst Appl. 2011; 38(5): 5809–5815. doi:10.1016/j.eswa.2010.10.050.

**6.** Piao Y, Piao M, Park K, Ryu KH. An ensemble correlation-based gene selection algorithm for cancer classification with gene expression data. Bioinformatics. 2012; 28(24): 3306–3315. doi:10.1093/bioinformatics/bts602.

**7.** Elyasigomari V, Lee DA, Screen HRC, Shaheed MH. Development of a two-stage gene selection method that incorporates a novel hybrid approach using the cuckoo optimization algorithm and harmony search for cancer classification. Journal of Biomedical Informatics. 2017; 67:11–20. doi:10.1016/j.jbi.2017.01.016.

**8.** Sampathkumar A, Rastogi R, Arukonda S, Shankar A, Kautish S, Sivaram M. An efficient hybrid methodology for detection of cancer-causing gene using CSC for micro array data. J Ambient Intell Humaniz Comput. 2020; 11(3):4743–4751. doi:10.1007/s12652-020-01731-7.

**9.** Pesaranghader A, Matwin S, Sokolova M, Beiko RG. SimDEF: definition-based semantic similarity measure of gene ontology terms for functional similarity analysis of genes. Bioinformatics 2016; 32(9): 1380–1387. doi: 10.1093/bioinformatics/btv755.

**10.** Angerer P, Fischer DS, Theis FJ, Scialdone A, Marr C. Automatic identification of relevant genes from low-dimensional embeddings of single-cell RNA-seq data. Bioinformatics. 2020;36(15):4291–4295. doi: 10.1093/bioinformatics/btaa198.

**11.** Kuang S, Wei Y, Wang L. Expression-based prediction of human essential genes and candidate lncRNAs in cancer cells. Bioinformatics. 2021; 37(3):396–403. doi:10.1093/bioinformatics/btaa717.

**12.** Zeng XQ, Li GZ, Yang JY, Yang MQ, Wu GF. Dimension reduction with redundant gene elimination for tumor classification. BMC Bioinformatics 2008; 9 (Suppl 6): S8. doi:10.1186/1471-2105-9-S6-S8.

**13.** Ono H, Ishii K, Kozaki T, Ogiwara I, Kanekatsu M, Yamada T. Removal of redundant contigs from de novo RNA-Seq assemblies via homology search improves accurate detection of differentially expressed genes. BMC Genomics. 2015; 16(1):1031–1044. doi: 10.1186/s12864-015-2247-0.

**14.** Suyan T. Identification of subtypespecific prognostic signatures using Cox models with redundant gene elimination. Oncology Letters. 2018; 15:8545-8555. doi: 10.3892/ol.2018.8418.

**15.** Pashaei E, Aydin N. Binary black hole algorithm for feature selection and classification on biological data. Applied Soft Computing. 2017;56,94–106. doi: 10.1016/j.asoc.2017.03.002.

**16.** Xiao Y, Hsiao T-H, Suresh U, Chen H-IH, Wu X, Wolf SE, et al. A novel significance score for gene selection and ranking. Bioinformatics. 2014;30(6):801–807. doi:10.1093/bioinformatics/btr671.

**17.** Deng H, Runger G.. Gene selection with guided regularized random forest. Pattern Recognition. 2013; 46(12): 3483–3489. doi: 10.1016/j.patcog.2013.05.018.

**18.** Alikovi E, Subasi A. Breast cancer diagnosis using GA feature selection and Rotation Forest. Neural Computing and Applications. 2017;28(4):753–763. doi: 10.1007/s00521-015-2103-9.

**19.** Subramanian A, Kuehn H, Gould J, Tamayo P, Mesirov JP. GSEA-P: a desktop application for gene set enrichment analysis. Bioinformatics. 2007; 23(23):3251–3253. doi:10.1093/bioinformatics/btm369.

**20.** Ogata H, Goto S, Sato K, Fujibuchi w, Bono H, Kanehisa M. KEGG: kyoto Encyclopedia of Genes and Genomes. Nucleic Acids Research. 1999;27(1):29–34. doi:10.1093/nar/27.1.29.

**21.**Liberzon A, Subramanian A, Pinchback R, Thorvaldsdóttir H, Tamayo p, Mesirov JP. Molecular signature database (msigdb) 3.0. Bioinformatics. 2011; 27(12):1739–1740. doi:10.1093/bioinformatics/btr260.

**22.** Reimand J, Isserlin R, Voisin V, Kucera M, Tannus-Lopes C, Rostamianfar A, et al. Pathway enrichment analysis and visualization of omics data using g:Profiler, GSEA, Cytoscape and EnrichmentMap. Nature Protocols. 2019; 14(2): 482–517. doi:10.1038/s41596-018-0103-9.

**23.** Robinson D. The statistical evaluation of medical tests for classification and prediction by m. sullivan pepe. Appl Stat. 2010;169(3): 656–656. doi: 10.1111/j.1467-985X.2006.00430_9.x

**24.** Khoury P, Gorse D. Investing in emerging markets using neural networks and particle swarm optimisation. International Joint Conference on Neural Networks. IEEE. 2015;1-7. doi:10.1109/IJCNN.2015.7280777.

**25.** Boughorbel S, Jarray F, El-Anbari M. Optimal classifier for imbalanced data using Matthews correlation coefficient metric. PLoS One. 2017;12(6):e0177678. doi: 10.1371/journal.pone.0177678.

**26.** Chawla NV, Karakoulas G. Learning from labeled and unlabeled data: an empirical study across techniques and domains. Journal of Artificial Intelligence Research. 2005; 23:331–366. doi:10.1613/jair.1509.

**27.** Fawcett T. An introduction to ROC analysis. Pattern Recognit Lett. 2006; 27(8): 861–874.

**28.** John GH, Kohavi R, Pfleger K. Irrelevant Features and the Subset Selection Problem. Machine Learning Proceedings 1994; 121–129. doi: 10.1016/B978-1-55860-335-6.50023-4.

**29.** Chen T, Guestrin C. XGBoost: a scalable tree boosting system. In: the proceedings of 22nd ACM SIGKDD conference on knowledge discovery and data mining. ACM.New York’ KDD. 2016; 785–794.

**30.** Geurts P, Ernst D. Wehenkel L. Extremely randomized trees. Machine Learning. 2006;63(1):3–42. doi: 10.1007/s10994-006-6226-1.

**31.** Foldvary N, Nashold B, Mascha E, Thompson EA, Lee N, McNamara JO, et al. Seizure outcome after temporal lobectomy for temporal lobe epilepsy: a kaplan-meier survival analysis. Neurology. 2000; 54(3):630–634. doi: 10.1212/WNL.54.3.630.

**32.** Singh D, Febbo PG, Ross K, Jackson DG, Manola J, Ladd C, et al. Gene expression correlates of clinical prostate cancer behavior, Cancer Cell. 2002;1(2):203–209. doi:10.1016/S1535-6108(02)00030-2.

**33.** Alon U, Barkai N, Notterman DA, Gish K, Ybarra S, Mack D, et al. Broad patterns of gene expression revealed by clustering analysis of tumor and normal colon tissues probed by oligonucleotide arrays. Proc. Natl. Acad. Sci. USA. 1999; 96 (12): 6745–6750. doi:10.1073/pnas.96.12.6745.

3. **Add mathematical description of the methods covered. Algorithm are messy, please make them clear and easy to understand.**

**Response:** I am very sorry. I'm afraid I don't have this ability yet, but I will work hard in the future.
