# Supplementary material for: GSEA–SDBE: A gene selection method for breast cancer classification based on GSEA and analyzing differences in performance metrics
Source: PLoS One. 2022 Apr 26;17(4):e0263171. doi: 10.1371/journal.pone.0263171 (PMC9041804; doi:10.1371/journal.pone.0263171)
Supplement: Attachment — Submitted filename: Response to Reviewers.docx [file pone.0263171.s012.docx]

**Dear Editors and Reviewers:**

Thank you for your letter and for the reviewers’ comment concerning my manuscript entitled “GSEA–SDBE: A gene selection method for breast cancer classification based on GSEA and analyzing differences in performance metrics” (ID: PONE-D-21-09636). Those comments are all valuable and very helpful for revising and improving my paper, as well as the important guiding significance to my researches. I have studied comments carefully and have made correction which I hope meet with approval. Revised portion are marked in red in the paper. The main concerns in the paper and the responds to the reviewer’s comments are as flowing:

**Responds to the reviewer’s commants:**

**Reviewer #2:**

**1. Response to comment:** (The article is lacking an academic way of writing. It needs a thorough revision. Improve language and presentation of the paper).

**Response:** I am very sorry for my incorrect writing. So, I have seriously corrected the language. Please check the revised paper.

**2. Response to comment:** (Please consider various breast cancer datasets for showing the efficacy of the method)

**Response:** I am very sorry for my negligence. To show the efficacy of Pre-SDBE method, the classification performance of this method was compared with that of the three advanced algorithms for five cancer datasets (breast, lung, liver, colon, and prostate cancer datasets).

~~Performance comparison with other models~~

~~The Pre-SDBE is a simplified version of the GSEA-SDBE method, which does not contain the use of GSEA to filter out genes weakly associated with or unrelated to cancer. The other three state-of-the-art-methods [7], the genetic algorithm (GA), the particle swarm optimization (PSO) algorithm, and the cuckoo optimization algorithm and harmony search (COA-HS), also does not filter out DEGs that have no biological significance for cancer. Therefore, the performance of the Pre-SDBE can be compared with these three algorithms which use relevant genes selected by the minimum redundancy and maximum relevance (MRMR) as input data.~~

~~In the Pre-SDBE algorithm, the top 50 relevant genes from DEGs were selected in the step that selected genes by their importance based on a random forest model (S1 Fig). Next, these selected genes were fed into SDBE algorithm to pick the best genes while maintaining the highest accuracy. The number of iterations in SDBE algorithm was respectively set at 3 and 10 for the colon and prostate cancer dataset (S5 Table).~~

**~~Table 5. Information on the microarray datasets used in this comparison~~**

| **~~Microarray dataset~~** | **~~Number of genes~~** | **~~Number of samples~~** | **~~Normal~~** | **~~Tumor~~** |
| --- | --- | --- | --- | --- |
| ~~Prostate [32]~~ | ~~12,600~~ | ~~102~~ | ~~50~~ | ~~52~~ |
| ~~Colon [33]~~ | ~~7,457~~ | ~~62~~ | ~~22~~ | ~~40~~ |

~~In the case of the colon cancer dataset, the Pre-SDBE method outperformed other algorithms, reaching 100% accuracy with the fewest number of genes (2 genes) compared to all other algorithms (S2 Fig,). For the prostate cancer dataset, the Pre-SDBE method could also achieve accuracy (98.99%) with the fewest genes (5 genes) (Table 6 and S3 Fig).~~

**~~Table 6. Performance comparison of Pre-SDBE with three models for two microarray datasets~~**

| **~~Algorithm~~** | **~~Colon~~** | | | | **~~Prostate~~** | | | |
| --- | --- | --- | --- | --- | --- | --- | --- | --- |
|  | **~~#Genes~~** | **~~AC~~** | **~~SE~~** | **~~SP~~** | **~~#Genes~~** | **~~AC~~** | **~~SE~~** | **~~SP~~** |
| ~~Pre-SDBE~~ | ~~2~~ | ~~1.0000~~ | ~~1.0000~~ | ~~1.0000~~ | ~~5~~ | ~~0.9899~~ | ~~0.9899~~ | ~~0.9899~~ |
| ~~PSO [7]~~ | ~~11~~ | ~~0.9642~~ | ~~0.8580~~ | ~~1.0000~~ | ~~19~~ | ~~0.9804~~ | ~~0.9180~~ | ~~1.0000~~ |
| ~~GA1 [7]~~ | ~~14~~ | ~~0.9516~~ | ~~0.8460~~ | ~~1.0000~~ | ~~28~~ | ~~0.9804~~ | ~~0.9180~~ | ~~1.0000~~ |
| ~~COA-HS [7]~~ | ~~5~~ | ~~1.0000~~ | ~~1.0000~~ | ~~1.0000~~ | ~~5~~ | ~~1.0000~~ | ~~1.0000~~ | ~~1.0000~~ |

~~Pre-SDBE: A simplified version of the GSEA-SDBE method; AC: Accuracy; SE: Sensitivity; SP:Specificity; #Genes: Number of selected gene~~

**Performance comparison of GSEA–SDBE with that of other models**

To test the feature selection performance of the GSEA–SDBE method, a simplified version, named Pre-SDBE, which does not use GSEA to filter out genes weakly associated with or unrelated to cancer, was used.

The three advanced gene selection algorithms were the genetic algorithm (GA), particle swarm optimization (PSO) algorithm, and cuckoo optimization algorithm and harmony search (COA-HS). These algorithms use 100 relevant genes selected via the minimum redundancy and maximum relevance (MRMR) as input data and the SVM as a classifier [7].

The classification performance of Pre-SDBE was compared with that of the three advanced algorithms for five cancer datasets composed of DEGs in breast, lung, and liver cancers and genes expressed in prostate and colon cancers (Table 5).

**Table 5. Information on the datasets used for performance comparison.**

| **Name** | **Data sources** | **#Genes** | **#DEGs** | **#Samples** | **Normal** | **Tumor** |
| --- | --- | --- | --- | --- | --- | --- |
| **Breast** | TCGA ^a^ | 56,536 | 4,579 | 1,222 | 113 | 1,109 |
| **Lung** | TCGA ^a^ | 56,536 | 7,483 | 1,146 | 108 | 1,038 |
| **Liver** | TCGA ^a^ | 56,536 | 8,772 | 465 | 58 | 407 |
| **Prostate** | Microarray dataset ^b^ | 12,600 | － | 102 | 50 | 52 |
| **Colon** | Microarray dataset ^c^ | 7,457 | － | 62 | 22 | 40 |

^a^ Database (<https://cancergenome.nih.gov/>); ^b^ Singh et al. [44]; ^c^ Alon et al. [45];

#Genes: number of genes; #DEGs: number of differentially expressed genes (obtained using wilcox.tes with logFC >1 and FDR <0.05); #Samples: number of selected samples.

In the step of the Pre-SDBE algorithm selecting genes by their importance, the top 50 relevant genes were selected based on a random forest model (S1 Fig). Next, these genes were fed into the SDBE algorithm to identify the most relevant genes with the highest accuracy. The number of iterations in the SDBE algorithm was set at 6, 7, 23, 3, and 10 for the breast, lung, liver, colon, and prostate cancer datasets, respectively. The Fitness of PSO, GA, and COA-HS over 100 iterations for each cancer dataset are shown in S2 Fig.

Table 6 shows that for unbalanced data (breast, lung, and liver cancers), the classification metrics (MCCs) of PSO, GA, and COA-HS algorithms were much lower than those of Pre-SDBE (98.07, 97.45, and 96.98 for breast, lung, and liver cancers, respectively). This indicated that the PSO, GA, and COA-HS algorithms did not perform well for unbalanced data.

For the five cancer datasets, whether the data were balanced or unbalanced, Pre-SDBE outperformed the other three algorithms, achieving the highest classification accuracy while identifying fewer number of genes (Table 6). More details are shown in S3 Fig, S5 and S6 Tables.

**Table 6. Classification metrics of four optimization algorithms for five cancer datasets.**

| **Algorithm** | **Breast** | | | | | | **Lung** | | | | | |
| --- | --- | --- | --- | --- | --- | --- | --- | --- | --- | --- | --- | --- |
|  | **#Genes** | **MCC** | **RA** | **F1** | **SE** | **SP** | **#Genes** | **MCC** | **AUC** | **F1** | **SE** | **SP** |
| **Pre-SDBE** | 4 | 98.07 | 99.42 | 99.82 | 99.73 | 99.12 | 3 | 97.45 | 98.93 | 99.76 | 99.71 | 98.15 |
| **PSO** ^a^ | 30 | 82.98 | 95.56 | 98.18 | 97.00 | 94.12 | 29 | 88.29 | 98.72 | 98.70 | 97.44 | 100 |
| **GA** ^a^ | 18 | 88.87 | 98.80 | 98.78 | 97.60 | 100 | 15 | 90.88 | 99.04 | 99.03 | 98.08 | 100 |
| **COA-HS** ^a^ | 11 | 90.93 | 97.78 | 99.09 | 98.50 | 97.06 | 8 | 89.56 | 98.88 | 98.87 | 97.76 | 100 |

| **Liver** | | | | | | **Colon** | | | | **Prostate** | | | |
| --- | --- | --- | --- | --- | --- | --- | --- | --- | --- | --- | --- | --- | --- |
| **#Genes** | **MCC** | **RA** | **F1** | **SE** | **SP** | **#Genes** | **AC** | **SE** | **SP** | **#Genes** | **AC** | **SE** | **SP** |
| 3 | 96.98 | 98.12 | 99.63 | 99.75 | 96.49 | 2 | 100 | 100 | 100 | 5 | 98.99 | 98.99 | 98.99 |
| 24 | 62.03 | 91.87 | 91.15 | 83.74 | 100 | 11^a^ | 96.42^a^ | 85.80^a^ | 100^a^ | 19^a^ | 98.04^a^ | 91.80^a^ | 100^a^ |
| 16 | 68.30 | 93.90 | 93.51 | 87.80 | 100 | 14^a^ | 95.16^a^ | 84.60^a^ | 100^a^ | 28^a^ | 98.04^a^ | 91.80^a^ | 100^a^ |
| 9 | 72.73 | 95.12 | 94.87 | 90.24 | 100 | 5^a^ | 100^a^ | 100^a^ | 100^a^ | 5^a^ | 100^a^ | 100^a^ | 100^a^ |

^a^ Elyasigomari et al. [7]; Pre-SDBE: simplified version of the GSEA–SDBE method; RA: ROC_AUC_score; F1: F1_score; AC: accuracy; SE: sensitivity; SP: specificity; #Genes: number of selected genes.

Note: For unbalanced (breast, lung, and liver) and balanced data (colon and prostate), the performance metrics of the model are different.

**3. Response to comment:** (Add redundancy assessment measures in addition to MCC.)

**Response:** I'm very sorry. I can't find other redundancy assessment measures beyond of MCC, but I will work hard in the future.

**4. Response to comment:** (Provide biological significance of the selected genes by various methods.)

**Response:** Biological significance of the selected genes as followers.

**Relevance of the selected genes to cancer**

*VEGF-D* induces the formation of lymphatics within tumors, thereby facilitating the spread of the tumor to lymph nodes, and promotes tumor angiogenesis and growth [32–36]. *TSLP* is an interleukin-7 (IL-7)-like cytokine that is involved in the progression of various cancers and is a key mediator of breast cancer progression [37–40]. Human *PKMYT1* is an important regulator of the G2/M transition in the cell cycle. Studies have demonstrated that *PKMYT1* might be a therapeutic target in hepatocellular carcinoma and neuroblastoma [41–43].

**5. Response to comment:** (Provide experimental setup for the analysis in more details. What were the criteria used for parameter selection.)

**Response:** I am very sorry for my negligence. More details were added for the SDBE algorithm, as shown below.

**Stage 7:** The genes of the list *NG* were analyzed by backward elimination. At each step of backward elimination, the 10-fold classification mode, e.g., random forest (RF), support vector machine (SVM), k-nearest neighbor (KNN), extreme gradient boosting (XGBoost), and extremely randomized trees (ExtraTrees), and ExtraTrees, was trained and tested to calculate various performance metrics. After each step of backward elimination, the performance metrics were respectively added to the corresponding metric lists. Then, the iteration was terminated and the data were saved. However, if the number of iterations set based on experience was not reached, the metrics lists, which were respectively transposed, and the list *NG* were sent to stage 3 to start a new iteration.

**Stage 8:** Mapping analysis of the metrics lists and the list NG was performed and the smallest set of relevant genes needed to achieve the required sample classification performance was determined.
